# Polysialic and colanic acids metabolism in *Escherichia coli* K92 is regulated by RcsA and RcsB

**DOI:** 10.1042/BSR20130018

**Published:** 2013-05-24

**Authors:** Nicolás Navasa, Leandro Rodríguez-Aparicio, Miguel Ángel Ferrero, Andrea Monteagudo-Mera, Honorina Martínez-Blanco

**Affiliations:** Departamento de Biología Molecular, Área de Bioquímica y Biología Molecular, Universidad de León, Campus de Vegazana, 24071 León, Spain

**Keywords:** Capsular polysialic acid, colanic acid, qRT–PCR, RcsA and RcsB regulation, CA, colanic acid, *C*_T_, threshold cycle value, Glc–Pro, glucose–proline, LA, LB supplemented with 2% w/v agar, LB, Luria–Bertani, MM, minimal media, PA, polysialic acid, qRT–PCR, quantitative real-time PCR, RT–PCR, reverse transcriptase–PCR, Xyl-Asn, xylose-asparagine.

## Abstract

We have shown previously that *Escherichia coli* K92 produces two different capsular polymers known as CA (colanic acid) and PA (polysialic acid) in a thermoregulated manner. The complex Rcs phosphorelay is largely related to the regulation of CA synthesis. Through deletion of *rscA* and *rscB* genes, we show that the Rcs system is involved in the regulation of both CA and PA synthesis in *E. coli* K92. Deletion of either *rcsA* or *rcsB* genes resulted in decreased expression of *cps* (CA biosynthesis cluster) at 19°C and 37°C, but only CA production was reduced at 19°C. Concerning PA, both deletions enhanced its synthesis at 37°C, which does not correlate with the reduced *kps* (PA biosynthesis cluster) expression observed in the *rcsB* mutant. Under this condition, expression of the *nan* operon responsible for PA catabolism was greatly reduced. Although RcsA and RcsB acted as negative regulators of PA synthesis at 37°C, their absence did not reestablish PA expression at low temperatures, despite the deletion of *rcsB* resulting in enhanced *kps* expression. Finally, our results revealed that RcsB controlled the expression of several genes (*dsrA*, *rfaH*, *h-ns* and *slyA*) involved in the thermoregulation of CA and PA synthesis, indicating that RcsB is part of a complex regulatory mechanism governing the surface appearance in *E. coli*.

## INTRODUCTION

**Figure 1:**
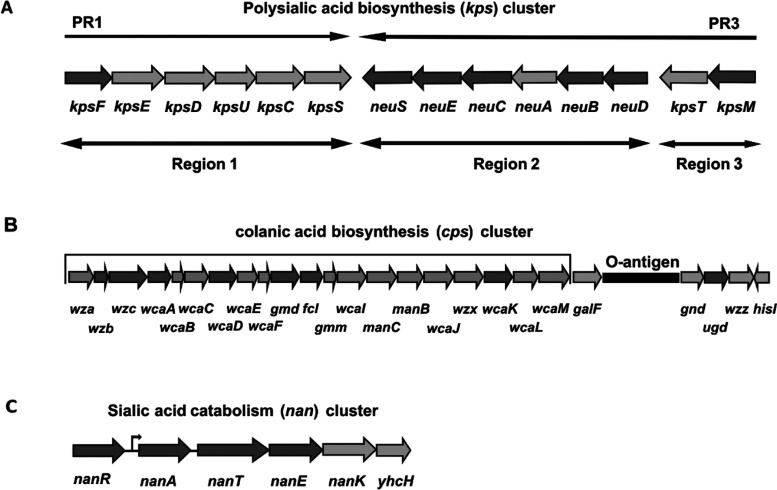
Genetic organization of *E. coli* PA and CA metabolism clusters (A) PA synthesis (*kps*), (B) CA synthesis (*cps*) and (C) PA catabolism (*nan*). Dark arrows indicate the genes used in the present study. PR1 and PR3: promoters located upstream of regions 1 and 3 in the *kps* cluster.

Over 80 distinct capsular or K antigens have been described in *Escherichia coli*, which are classified into four groups [1]. Group II K antigens exhibit capsule expression at 37°C but not at low temperatures (18°C) and their regulation is temperature-dependent [1,2]. The group II capsule gene cluster (*kps*) consists of a central serotype-specific region 2, encoding proteins for synthesis and polymerization of the specific K antigen that is flanked by conserved regions 1 and 3 (Figure 1A) [1,3]. Transcription of the *kps* cluster is driven by two convergent temperature-regulated promoters located upstream of regions 1 and 3 [4]. Transcription of regions 2 and 3 is driven by the PR3 promoter [4] and is dependent on RfaH for transcription elongation [5]. In addition, H-NS plays an unusual dual role, not only being required for maximal transcription at 37°C but also contributing to transcriptional repression at low temperatures (≤20°C) [4,6]. Transcription of region 1 is driven by promoter PR1, and its maximal expression at 37°C requires both H-NS and SlyA, whereas a reduced SlyA expression at 20°C results in repressed transcription from PR1 [4,6,7].

*E. coli* K92 synthesizes a type II capsule known as PA (polysialic acid), which is responsible for bacterial virulence [8,9]. As with the group II K antigens, PA is mainly generated at 37°C and is negligible at low temperatures (below 20°C) [10]. We previously showed that *E. coli* K92 is able to synthesize a different capsular polysaccharide known as CA (colanic acid) [11]. CA is associated with bacterial protection against desiccation, extreme temperatures and acidic environmental conditions [12,13], as well as against osmotic and oxidative stress [14]. In contrast to PA, CA is predominantly synthesized at low temperatures [11,15] and does not seem to play a role in bacterial virulence [1,16].

A complex signal transduction pathway, namely the Rcs phosphorelay, controls the expression of the *cps* operon (Figure 1B) responsible for CA production. This pathway can be activated at low temperatures and involves a histidine kinase (RcsC), a response regulator (RcsB), a phospho-transfer protein (RcsD), a signal transductor (RcsF) and an auxiliary activator protein (RcsA) [15,17]. The transcriptional activator RcsB is the principal regulator of this system and it forms homodimers which activate *cps* operon transcription. In addition, RcsB may form heterodimers with the auxiliary activator RcsA, enhancing *cps* operon transcription. It has been described that RcsB is required for *cps* gene expression, whereas the absence of RcsA only decreases it [15]. Under normal conditions, the amount of RcsA protein is limited by its low synthesis level and because it is rapidly degraded by a Lon protease. However, synthesis of RcsA is increased at low temperatures, leading to a mucoid phenotype as a consequence of the expression of *cps* genes [15]. In addition, other regulatory molecules are implicated, either directly or indirectly, in control of CA synthesis. Thus, the anti-termination factor RfaH is required for transcription of genes downstream of *wzc* [5,18], while DsrA is a small molecule of RNA which increases *cps* transcription through its negative regulation of H-NS synthesis [19].

Although most of the targets present in the Rcs regulon are positively regulated, it has been suggested that the Rcs regulon also may repress the synthesis of type II capsules in *E. coli* [20]. Through deletion of *rcsB* and *rcsA* genes, in this report we show that RcsA and RcsB act as negative regulators of PA synthesis at 37°C in *E. coli* K92, although the mechanism remains unclear. Moreover, both proteins enhance the expression of the *cps* operon, which is required for maximal CA production at low temperatures. However, they barely play a role in CA synthesis at high temperatures (37°C). More importantly, neither *rcsA* nor *rcsB* deletion completely abolished CA synthesis under any of the conditions tested. In sum, these data suggest that the Rcs system is a critical regulator of the adaptation response of *E. coli* K92 to different environmental conditions through the expression of PA and CA capsules.

## MATERIALS AND METHODS

### Strains, culture media and growth conditions

The strains and plasmids used in the present study are shown in Table 1. Bacterial cultures were inoculated and grown at 37 or 19°C as previously described in Navasa et al. [21]. Bacterial cultures were grown in LB (Luria–Bertani) complex medium, LA [LB supplemented with 2% (w/v) agar] and Xyl–Asn (xylose–asparagine) or Glc–Pro (glucose–proline) MM (minimal media) for *E. coli* [22]. Where indicated, Glc–Pro MM was supplemented with agar 2% (w/v). We chose Xyl–Asn or Glc–Pro MM because they induce maximal PA and CA production in *E. coli* K92, respectively [2,14]. During the gene allelic exchange experiments, LA medium supplemented with 5% (w/v) sucrose and without NaCl was used to select plasmid excision from the chromosome [23]. When required, the following supplements were added to the culture media: rifampicin (25 and 10 μg/ml for liquid and solid media, respectively), kanamycin (25 and 12.5 μg/ml for liquid and solid media, respectively), ampicillin (100 μg/ml) and chloramphenicol (60 μg/ml).

**Table 1:** Strains and plasmids used in the present study

(a) *E. coli* strains		
Strain	Description	Reference or source
DH5α’	F^−^*Δlac*U169 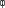 80d*lac*Z1M15 *hsd*R17 *rec*A1 *end*A1 *gyr*A96 *thy*-1 λ^−^*rel*A1 *sup*E44 *deo*R	[46]
S17-1λpir	RP4 2-Tc::Mu-Km::Tn*7prothirecA*HsdR^−^M^+^λ*pir*	[29]
K92	Wild-type	ATCC 35860
K92ΔrcsA	K92 *ΔrcsA*; constructed using pDS132-WX	The present study
K92ΔrcsB	K92 *ΔrcsB*; constructed using pDS132-YZ	The present study
(b) Plasmids and constructions		
Plsmid		
pGEM-T Easy	Ap^r^*ori*ColE1 *lac*Zα^+^ SP6 T7 *lac* promoter, direct cloning of PCR products	Promega
pDS132	R6K *ori mob*RP4 *cat sacB*	[23]
pGEM-W	*rcsA* upstream sequences PCR amplified with primers rcsAup5′ and rcsAup3 cloned into pGEMT-easy; Ap^r^	The present study
pGEM-X	*rcsA* downstream sequences PCR amplified with primers rcsAdown5′ and rcsAdown3 cloned into pGEMT-easy; Ap^r^	The present study
pGEM-Y	*rcsB* upstream sequences PCR amplified with primers rcsBup5′ and rcsBup3 cloned into pGEMT-easy; Ap^r^	The present study
pGEM-Z	*rcsB* downstream sequences PCR amplified with primers rcsBdown5′ and rcsBdown3′ cloned into pGEMT-easy; Ap^r^	The present study
pGEM-WX	*ΔrcsA*; *rcsA* upstream sequence from pGEM-W removed with EcoRI and ligated with *rcsA* downstream sequence from pGEM-X removed with EcoRI; Ap^r^	The present study
pGEM-YZ	*ΔrcsB*; *rcsB* upstream sequence from pGEM-Y removed with EcoRI and ligated with *rcsB* downstream sequence from pGEM-Z removed with EcoRI; Apr	The present study
pDS132-WX	*ΔrcsA* sequences from pGEM-WX removed with SacI and SphI inserted into pDS132 digested with the same enzymes; Cat^r^	The present study
pDS132-YZ	*ΔrcsB* sequences from pGEM-YZ removed with SacI and SphI inserted into pDS132 digested with the same enzymes; Cat^r^	The present study

### DNA manipulation and RNA isolation

Routine molecular biology techniques were performed according to standard procedures [24]. Restriction and modifying enzymes (Invitrogen S.A.) were used as recommended by the manufacturer. Plasmid DNA was isolated from *E. coli* using the Wizard R Plus SV Minipreps DNA Purification System (Promega). For deletion experiments, PCR products were generated by Taq DNA polymerase (Stratagene) using the *E. coli* K92 ATCC 35860 genome as the template and the primers described in Table 2. *E. coli* cells were transformed by the method described by Donnenberg and Kaper [25]. Mobilization of plasmids between *E. coli* strains was accomplished as described previously [26]. Purification of total RNA was performed using an Illustra RNAspin Mini RNA Isolation Kit (GE Healthcare). The isolated total RNA was treated with DNAse I (Invitrogen S.A.) and quantified by spectrophotometry [24].

**Table 2:** Primers used in the present study

Function	Name	Sequence (5′→3′)
*rcsA* deletion	rcsAup5′	CGACTAGGTTTAACCGGGTATCTG
	rcsAup3′	GTTGATTAATGATGAGCTTGATACGC
	rcsAdown5′	CGACGTTATCATTGAGCCGAAC
	rcsAdown3′	CATTAGTCACATTATCCGTCAGTCG
*rcsB* deletion	rcsBup5′	TGAACGTAATTATTGCCGATGACC
	rcsBup3′	GGAAATGGCGCTTGATGTACTTG
	rcsBdown5′	CAGTGCTGGTGGTTACGGTGAC
	rcsBdown3′	CTTTATCTGCCGGACTTAAGGTCAC

### qRT–PCR (quantitative real-time PCR)

The DNase-treated RNA was reverse transcribed with the ThermoScript™ RT–PCR (reverse transcriptase–PCR) System (Invitrogen S.A.). For qRT–PCR, each cDNA product was used as a template for DNA amplification, using primer pairs as previously described [21], 10 μl of SYBR® Green PCR Master Mix (Applied Biosystems) and up to 20 μl of water. Primers were designed using the Oligo Primer Analysis Software [27], based on sequences retrieved from the GenBank/EMBL databases. In all cases, the oligonucleotides used in qPCR (quantitative PCR) were designed to have similar melting temperatures (60°C) and to amplify DNA fragments of similar lengths (around 100 nucleotides). Reactions were performed using an ABI Prism 7000 sequence detection system (Applied Biosystems) and applying the following conditions: 50°C for 2 min, 95°C for 5 min, 40 cycles of 94°C for 15 s and 60°C for 1 min. The results were processed using specific software (ABI Prism 7000 SDS software). The relative gene expression levels were calculated as previously described [28] using the equation: Δ*C*_T_=(*C*_T_ gene at x°C, Y genotype−*C*_T_ housekeeping gene at x°C, Y genotype) − (*C*_T_ gene at x°C, WT (wild-type) genotype − *C*_T_ housekeeping gene at x°C, WT genotype) and then transformed into relative changes (*n*-fold) using 2^−ΔΔ*C*^_T_. *C*_T_ (threshold cycle value) is the cycle number at which the real-time amplification curve crosses the user-defined threshold, x°C is the temperature at which the RNA was isolated (37°C or 19°C) and Y is the mutant strain (*E. coli K92ΔrcsA* or *E. coli K92ΔrcsB*). The data represent the average change (*n*-fold) determined from at least three independent experiments. As a control we used the housekeeping gene *gapdh*, which was carefully validated before its use in the quantitative mRNA assays, with 16S rRNA expression as internal control obtained under the same conditions and determined from at least three independent experiments.

### *sacB*-assisted allelic exchange mutagenesis

pDS132-based allelic exchange plasmids [23] were electroporated into *E. coli* S17-1λ*pir* cells for conjugation into *E. coli* K92 by filter mating [29]. Transconjugants containing single crossovers of the allelic exchange plasmid integrated into the *E. coli* K92 genome were selected in LA supplemented with chloramphenicol and rifampicin. To force the second recombination, single-crossover strains were plated onto LA containing rifampicin and 5% (w/v) sucrose and then incubated at 37°C for 24–48 h. Sucrose-resistant colonies were placed on LA-sucrose plates and screened for a loss of chloramphenicol resistance encoded by the vector.

### Deletion of *rcsA* and *rcsB* genes from *E. coli* K92

The 0.3-kb upstream and downstream sequences included in both *rcsA* or *rcsB* loci were PCR amplified using the primers indicated in Table 2. These 0.3-kb amplicons were individually cloned into pGEM-T Easy, yielding plasmids pGEM-W, pGEM-X, pGEM-Y and pGEM-Z, respectively. The cloned sequences were excised using EcoRI. Downstream and upstream sequences for each gene were ligated and the products were amplified using the primer pairs rcsAup5′ and rcsAdown3′ and rcsBup5′ and rcsBdown3′, and cloned again into pGEM-T Easy, yielding pGEM-WX and pGEM-YZ. The *rcsA* and *rcsB* upstream–downstream DNA sequences (*ΔrcsA* and *ΔrcsB*, respectively) were excised from pGEM-WX and pGEM-YZ using SacI and SphI restriction enzymes and cloned into pDS132 previously digested with the same enzymes, yielding plasmids pDS132-WX and pDS132-YZ, respectively. The suicide vectors carrying *ΔrcsA* or *ΔrcsB* were electroporated into *E. coli* S17-1*λpir* for biparental conjugation into *E. coli* K92. The deletions were recombined into the chromosome of *E. coli* K92 by using the standard two-step sucrose-resistance-assisted allelic exchange method described above. The correct allelic exchange of the WT allele for each mutant allele was confirmed by PCR using the primers rcsAup5′and rcsAdown3′, and rcsBup5′and rcsBdown3′. The *ΔrcsA* and *ΔrcsB E. coli* K92 mutants were named *E. coli* K92*ΔrcsA* and *E. coli* K92*ΔrcsB*, respectively. For each mutant, loss of the DNA fragment was confirmed by PCR and the absence of expression of the deleted gene was confirmed by qRT–PCR.

### Quantification of exopolysaccharides

Quantitative determination of CA and PA production by *E. coli* K92 cultures was performed as previously described [11]. Briefly, *E. coli* K92 cells were removed by centrifugation and the cell-free supernatant obtained was dialysed against 1000 vol of distilled water for 24 h at 4°C. Dialysed supernatant samples were used for quantitative determination of CA by the orcinol method [30], according to the amount of uronic acids. Dialysed supernatant samples were also used for quantitative determination of PA following the resorcinol protocol described by Svennerholm [31].

### Capsule staining

The presence or absence of CA surrounding the bacteria was evaluated by using a combination of negative and fuchsine staining procedures. Briefly, a very small drop of bacterial culture was placed near one end of a well-cleaned slide. Once dry, one drop of fuchsine (primary colorant) was added to the bacterial culture without spreading for 2 min. After that, the sample was washed with water and dried. Next, a drop of nigrosin (India ink) no greater than the drop of bacterial culture was added and the mixture was spread over the slide using another clean slide. Finally, a cover glass was placed on the sample, avoiding the formation of bubbles and examined under a Nikon Eclipse E600 optical microscope.

### Statistical analysis

The results are presented as means±S.E. Significant differences between means were calculated with Student's *t* test. *P* values of 0.05 or less were considered statistically significant.

## RESULTS

### *rcsA* and *rcsB* gene products control CA synthesis in *E. coli* K92 at low temperatures

**Figure 2:**
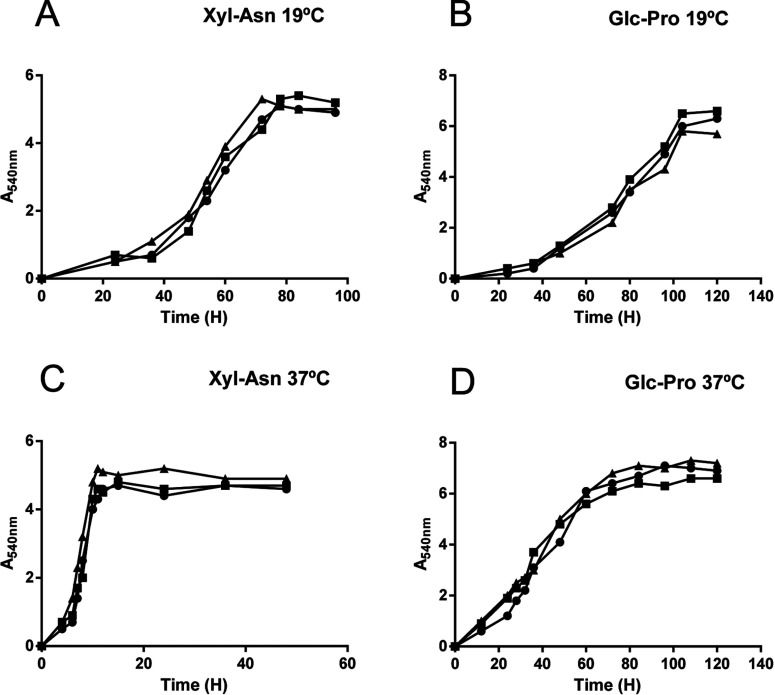
Bacterial growth of *E. coli* K92 (circle), *E. coli* K92*ΔrcsA* (square) and *E. coli* K92*ΔrcsB* (triangle) incubated in MM containing Xyl–Asn (A,C) or Glc-Pro (B,D)at 19°C (A,B) or 37°C (C,D)

**Figure 3:**
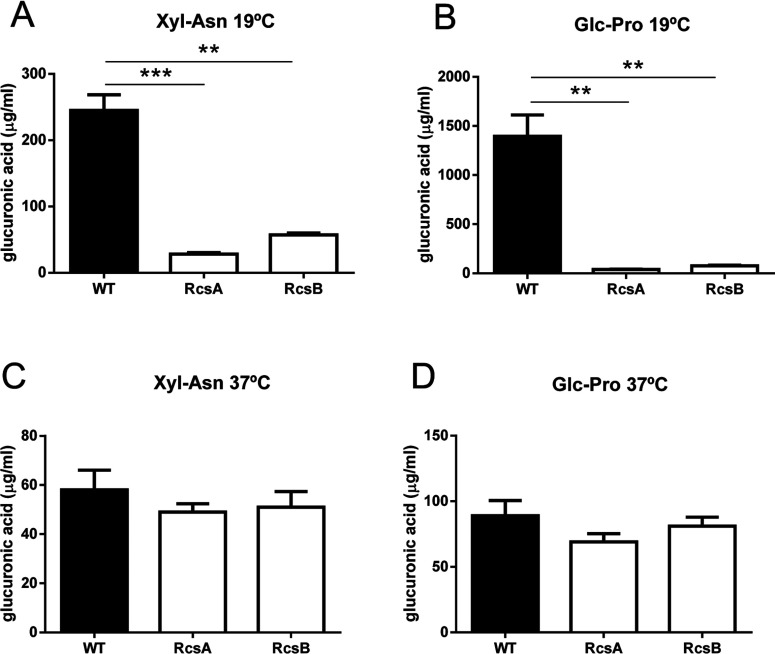
CA production (measured as glucuronic acid) by *E. coli* K92, *E. coli* K92*ΔrcsA* and *E. coli* K92*ΔrcsB* growth in MM containing Xyl–Asn (A,C) or Glc-Pro (B,D) at 19°C (A,B) and 37° (C,D) **P<0.005, ***P<0.001 by Student's *t* test.

**Figure 4:**
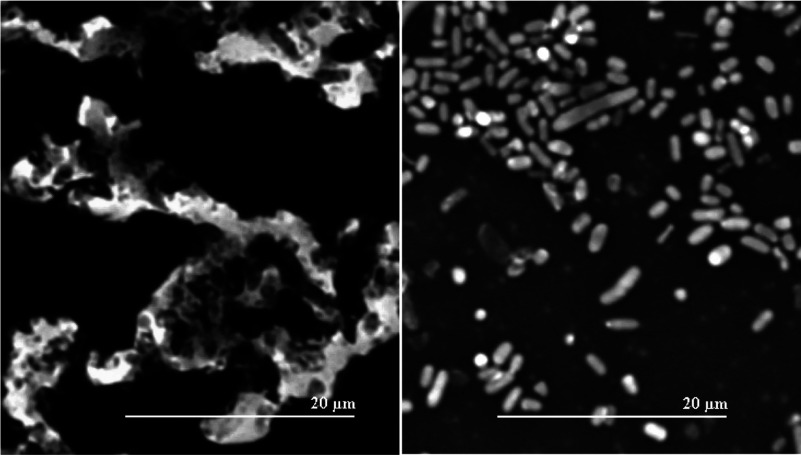
Microphotographs taken from *E. coli* K92 (left) and *E. coli* K92*ΔrcsA* (right) previously grown in MM containing Glc-Pro with agar at 19°C and visualized by Burri's method using China ink and optical microscope

We previously showed that *E. coli* K92 (WT) synthesizes CA as capsular polymers in a temperature-dependent manner [11,21]. It is well known that Rcs phosphorelay and the auxiliary protein RcsA act as positive regulators of CA synthesis [15]. To investigate the role of RcsA and RcsB in CA synthesis by *E. coli* K92, we performed gene deletion experiments to obtain *E. coli* K92*ΔrcsA* and *E. coli* K92*ΔrcsB* mutant strains lacking *rcsA* and *rcsB* genes, respectively. WT and both mutant strains were grown in Glc–Pro and Xyl–Asn MM at 19°C, the optimal growth temperature for synthesis of CA by *E. coli* K92 [11,12], and production of this polymer was determined after 120 h. Neither mutant showed any change in growth under the conditions tested (Figure 2) and both deletions resulted in a dramatically decreased CA production at 19°C (measured as glucuronic acid content) in the media tested (Figures 3A and 3B), but it was not completely abrogated. We ensured that all glucuronic acid detected belonged to a high molecular mass structure, such as CA polymer, through prior dialysis of supernatants using a 10 kDa membrane pore size. To assess the absence of CA capsules surrounding the bacteria, WT and *E. coli* K92*ΔrcsA* were grown on Glc–Pro MM agar plates at 19°C and the Burri method using China ink was applied. This staining technique revealed large amounts of capsular polymer produced by WT in contrast to the mutant strain (Figure 4).

Next, we examined whether RcsA and RcsB control CA production at 37°C. At this temperature, the levels of CA produced by both mutants were similar to those produced by WT (Figures 3C and 3D). Overall, these results indicate that both RcsA and RcsB positively regulate CA synthesis by *E. coli* K92 at low temperatures and suggest an Rcs phosphorelay-independent CA synthesis at high temperatures.

### Deletion of *rcsA* or *rcsB* genes down-regulates the expression of CA synthesis genes

To determine whether RcsA and RcsB regulate the expression of the *cps* operon responsible for CA synthesis, WT and both mutant strains were grown at 19°C to enhance CA production, and RNA samples harvested from mid-exponential phase were used to analyse the expression of several genes belonging to the *cps* operon (Figure 1B) by RT–PCR. Consistent with the reduced CA production, deletion of *rcsA* resulted in decreased *cps* gene expression of 1.4- and 8.5-fold (Table 3). This effect was greater in the absence of RcsB, resulting in a reduction of up to 35-fold. We also analysed expression of the *ugd* gene, which is located outside the *cps* operon but is also involved in CA synthesis [32]. Deletion of *rcsB* reduced *ugd* expression 2.3-fold, whereas deletion of *rcsA* barely increased it (1.4-fold).

**Table 3:** Expression level differences of CA metabolism genes between *E. coli* K92 and *E. coli* K92*ΔrcsA* or *E. coli* K92*ΔrcsB* measured by qPCR at 19 and 37°C

Function	Gene*	Product†	*E. coli K*92*ΔrcsA*/ *E. coli K*92‡ 19°C	*E. coli K*92*ΔrcsB*/ *E. coli K*92‡ 19°C	*E. coli* K92*ΔrcsB*/ *E. coli K*92‡ 37°C
CA synthesis	*wzb*	Tyrosine phosphatase	−1.4±0.2	−16.9±1.7	−2.1±0.3
	*wzc*	Tyrosine kinase	−1.7±0.2	−14.7±2.2	−2.7±0.3
	*wcaA*	Putative colanic acid glycosyltransferase	−2.7±0.3	−17.9±2.1	−2.3±0.4
	*wcaD*	Colanic acid polymerase	−6.9±0,9	−23.3±3.1	−3.1±0.5
	*gmd*	GDP-mannose 4,6-dehydratase	−8.5±1.3	−35.7±3.7	−28.8±4.8
	*fcl*	GDP-fucose synthase	−7.3±1.5	−29.7±3.1	−21.8±5.5
	*wcaK*	Putative colanic acid piruviltransferase	−6.8±0.8	−9.2±1.2	−4.9±1.1
	*ugd*	UDP-glucose-6-dehydrogenase	+1.4±0.2	−2.3±0.3	−3.3±0.2

*Genes involved in metabolism of CA.

†Description of the products encoded by genes.

‡The relative levels of gene expression were calculated as described in the Materials and methods, and then transformed to relative change using the formula 2^−ΔCT^. As a control gene we used the housekeeping gene *gapdh*.

Since deletion of *rcsB* resulted in a higher reduction in expression of *ugd* and *cps* genes at 19°C, we also investigated whether RcsB controls their transcription at 37°C, even though its absence barely decreased CA production at this temperature. Deletion of *rcsB* resulted in a lower decrease in *cps* gene expression (between 2.1 and 4.9-fold) (Table 3) than that observed at 19°C, and only the expression of *gmd* and *fcl* genes was reduced in a similar manner. We compared relative gene expressions for both temperatures given that temperature barely changed *cps* expression in *E. coli* K92 [21]. In addition, deletion of *rcsB* decreased *ugd* expression 3.3-fold.

### *rcsA* and *rcsB* gene products repress PA synthesis in *E. coli* K92

*E. coli* K92 predominantly synthesizes PA type II capsules at 37°C, and no production was detected below 20°C [2,10,21]. It has been suggested that the Rcs phosphorelay system may negatively regulate bacterial group II capsule synthesis [20]. To investigate this, WT and both mutant strains were grown in Glc–Pro and Xyl–Asn MM at 37°C, the optimal growth temperature for synthesis of PA by *E. coli* K92 [11,21], and the production of this polymer was measured after 120 h.

**Figure 5:**
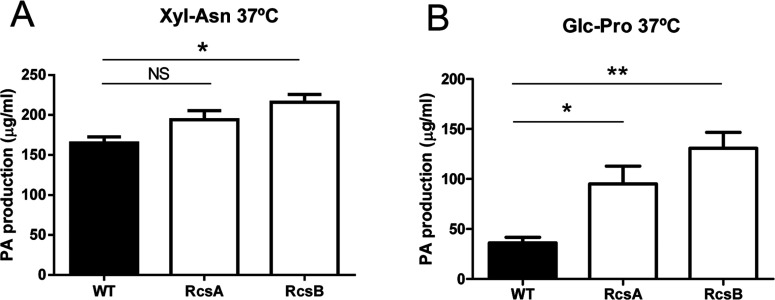
PA production by *E. coli* K92, *E. coli* K92*ΔrcsA* and *E. coli* K92*ΔrcsB* growth in MM containing Xyl–Asn (A) or Glc–Pro (B) at 37°C NS, no statistically significant differences; *P<0.05, **P=0.005 by Student's *t* test.

Deletion of either *rcsA* or *rcsB* resulted in increased PA production under all conditions tested (Figure 5). This increment was not significant when *E. coli* K92*ΔrcsA* was grown in Xyl–Asn MM. Dialysis of the supernatants ensured that all sialic acid detected belonged to a high molecular mass structure. These results show that RcsA and RcsB act, directly or indirectly, to repress PA synthesis in *E. coli* K92. However, this effect was insufficient to overcome inhibition of PA capsule synthesis at low temperatures, since neither *E. coli* K92*ΔrcsA* nor *E. coli* K92*ΔrcsB* were able to generate PA synthesis at 19°C (results not shown).

### Dual role of RcsB in transcriptional regulation of PA metabolism genes

We next investigated whether RcsB controls the expression of the *kps* operon responsible for PA synthesis. WT and *E. coli* K92*ΔrcsB* were grown at 19°C and 37°C, and RNA samples harvested from mid-exponential phase were used to analyse the expression of several genes belonging to the *kps* operon [1,10]. At 19°C, the deletion of *rcsB* resulted in an increase (between 1.4- and 7.7-fold) in the expression of all tested genes (Table 4), although no PA production was observed. In contrast, *E. coli* K92*ΔrcsB* showed a reduction (up to 2.3-fold) in *kps* gene expression at 37°C with respect to WT (Table 4), which does not correlate with the increased PA synthesis (see Figure 4). To determine whether increased PA production at 37°C could be due to a diminished PA catabolism, we also analysed the expression of several genes belonging to the *nan* (PA catabolism) operon (Figure 1C) and the regulator NanR. Interestingly, the expression of *nanAET* genes was greatly reduced at 37°C (up to 10-fold), suggesting a diminished PA catabolism, whereas *nanR* expression remained unchanged. Finally, deletion of *rcsB* resulted in a slight increase in the expression of *nan* genes at 19°C (Table 4).

**Table 4:** Expression levels differences of genes involved in metabolism and regulation of the PA between *E. coli* K92 and *E. coli* K92*ΔrcsB* measured by qPCR at 19°C and 37°C

Function	Gene*	Product†	*E. coli K*92*ΔrcsB*/*E. coli K*92‡ 19°C‡	*E. coli K*92*ΔrcsB*/*E. coli K*92‡ 37°C‡
Syalic acid and PA synthesis (from *kps* cluster)	*kpsF*	Putative transcriptional start site	+1.4±0.2	−2.0±0.3
	*neuB*	NeuNAc synthase	+5.6±0.8	−2.1±0.3
	*neuC*	UDP-GlcNAc epimerase	+2.9±0.3	−1.8±0.3
	*neuD*	Acyltransferase	+3.9±0.3	−1.3±0.1
	*neuE*	Sialic acid transport and polymerization	+6.4±1.0	−2.3±0.1
	*neuS*	Polysialyltransferase	+7.7±0.8	−1.9±0.3
	*kpsM*	ABC-transporter	+2.3±0.2	−1.1±0.0
Syalic acid metabolism and regulation (from *nan* system)	*nanA*	N-Acetyl neuraminatelyase	+1.4±0.1	−5.3±0.4
	*nanE*	N-Acetyl manosamine-6-P epimerase	+1.8±0.1	−10.6±1.1
	*nanT*	Sialic acid transporter	+1.7±0.2	−5.3±0.6
	*nanR*	Transcriptional dual regulator	+1.9±0.1	+1.0±0.0

*Genes involved in metabolism and regulation of PA.

†Description of the products encoded by genes.

‡The relative levels of gene expression were calculated as described in the Materials and methods, and then transformed to relative change using the formula 2^−ΔCT^. As a control gene we used the housekeeping gene *gapdh*

### RcsB as part of a complex network regulating both CA and PA synthesis

In order to gain a better understanding of the regulatory mechanism involved in the thermoregulated synthesis of CA and PA in *E. coli* K92, we further investigated whether RcsB controls the expression of several genes implicated in the regulation of CA and/or PA synthesis. Firstly, we analysed the expression of *rcsC, rcsF* and *rcsA* genes belonging to the Rcs pathway. Deletion of *rcsB* resulted in a greater reduction in *rcsA* expression at both temperatures compared with WT (29.5- and 128-fold at 19 and 37°C, respectively) (Table 5), consistent with the key role of RcsB in high-level expression of *rcsA* [33]. *rcsC* gene expression was up-regulated at 19°C (2.8-fold) but down-regulated at 37°C (3.5-fold) in the mutant strain, whereas *rcsF* expression remained unchanged.

**Table 5:** Expression level differences of genes involved in the regulation of the CA and/or PA between *E. coli* K92 and *E. coli* K92*ΔrcsB* measured by qPCR at 19°C or 37°C

Function	Gene*	Product†	*E. coli K*92*ΔrcsB*/*E. coli* K92‡ 19°C‡	*E. coli K*92*ΔrcsB*/*E. coli* K92‡ 37°C‡
CA regulation	*rcsA*	Transcriptional dual regulator	−29.5±3.5	−128.0±22.1
	*rcsC*	Sensor	+2.8±0.2	−3.5±0.3
	*rcsF*	Glucose zinc sensor	+1.0±0.0	−1.1±0.0
Regulation of thermoregulatory	*dsrA*	RNA h-ns riborregulator	+7.6±2.1	−1.4±0.1
polysaccharide synthesis	*rfaH*	Transcriptional antitermination factor	+2.0±0.1	−2.4±0.1
	*h-ns*	Transcriptional dual regulator	+1.5±0.3	−2.0±0.3
	*slyA*	Transcriptional activator	+1.4±0.1	−1.8±0.2

*Genes involved in metabolism and regulation of CA and/or PA.

†Description of the products encoded by genes.

‡The relative levels of gene expression were calculated as described in the Materials and methods, and then transformed to relative change using the formula 2^−ΔCT^. As a control gene we used the housekeeping gene *gapdh*.

We also analysed the expression of *dsrA*, *rfaH*, *h-ns* and *slyA* genes, which are temperature-dependent in *E. coli* K92 [21]. DsrA RNA positively regulates *rcsA* expression [15] and the anti-terminator RfaH is required for the expression of large polysaccharide clusters, including *kps* [5,34] and presumably *cps*, whereas H-NS and SlyA are required not only for maximum transcription of the *kps* operon at 37°C but also to repress it at 19°C [7]. Deletion of *rcsB* resulted in an increased expression of all four genes at 19°C, especially *dsrA* (7.6-fold), and a reduced expression at 37°C (between 1.4- and 2.4-fold).

## DISCUSSION

The mechanism by which the Rcs system promotes CA synthesis is well known. As a homodimer, or forming heterodimers with RcsA, RcsB binds to the RcsAB box to enhance *cps* transcription [34]. Consistent with this, we found a decreased *cps* expression in *rcsA* and *rcsB* mutants at both temperatures (Table 3). This effect was greater in the *rcsB* mutant at 19°C, probably because deletion of *rcsB* has a double effect, abrogating both RcsB- and RcsAB-mediated *cps* transcription. Thus, it has been described that RcsB is required as the main regulator for *cps* transcription and no expression is detected in its absence, whereas RcsA merely enhances it [15]. However, we found that *cps* expression was readily detectable at both temperatures, even in *rcsB* mutants. Since RcsA alone is unable to promote *cps* expression [21], these results suggest an RcsB-independent *cps* transcription. The *cps* cluster not only codifies enzymes involved in CA synthesis but also in other pathways. For example, Wzc not only participates in the polymerization and exportation of CA capsules [1] but also enhances Ugd activity through phosphorylation [35]. The *ugd* product is involved in many cellular processes such as capsular polysaccharide [36,37] and LPS (lipopolysaccharide) synthesis, polymyxin B resistance [38] and regulation of the levels of the sigma factor RpoS [39]. Thus, it would not be surprising that the regulation of Wzc was under the control of regulators other than RcsB, reflecting the variety of processes which are involved.

Several findings prompt us to believe that transcriptional regulation of the Rcs system on *cps* expression does not determine CA synthesis at high temperatures. Thus, reduced *cps* transcription in the *rcsB* mutant did not result in a reduced CA production at 37°C (Figures 3B and 3D). Although CA production at 37°C was lower than at 19°C, it cannot be considered as residual since values of CA production observed at 37°C were close to, for example, those observed in Xyl–Asn MM at 19°C, and in this case, CA production substantially decreased to a statistically similar degree in both *rcsA* and *rcsB* mutants (Figures 3A and 3C). In addition, we might expect that a higher expression of *rcsB* would result in an increased *cps* transcription, and consequently, in enhanced CA production; however, previous results showed that the *rcsB* gene expresses 6-fold higher at 37°C than at 19°C in WT [21]. Furthermore, the thermoregulation of CA production is not probably explained through changes in *cps* expression, whether or not the Rcs pathway is involved. This is supported by the fact that *E. coli* K92 produced large amounts of CA at 19°C in contrast to 37°C, but minimal changes in *cps* transcription were detected [21], suggesting that post-transcriptional modifications take place. In this sense, phosphorylation of Ugd by Wzc was found to participate in the regulation of CA production [35]. Furthermore, the phosphorylation/dephosphorylation cycle in tyrosine kinase Wzc was shown to control the production and size distribution of CA polymer in *E. coli* K-12 and, more importantly, an external desiccation signal was directly linked to the phosphorylation state [40]. Whether the Rcs pathway plays a role at the post-transcriptional level remains unknown.

Unlike CA synthesis, the thermoregulation of group II capsule synthesis in *E. coli* occurs at a transcriptional level [4,7,41]. Consistent with this, we previously observed that *kps* operon expression was down-regulated by up to 500-fold in *E. coli* K92 at low temperatures with respect to high temperatures [21]. Russo and Singh [20] have suggested that group II K54 capsule expression is negatively regulated by RcsA, and this effect appears to be mediated through RcsB. We found that deletion of either *rcsA* or *rcsB* enhanced *kps* transcription by up to approximately 8-fold at 19°C (Table 4). This effect is probably physiologically insignificant since *kps* expression at low temperatures is highly reduced [21]; however, it might reveal a role of the Rcs pathway negatively regulating *kps* transcription. We also found that deletion of either *rcsA* or *rcsB* resulted in an enhanced PA production at 37°C (Figure 5), but we failed to explain this effect through transcriptional regulation of the *kps* operon, whose expression was slightly reduced (Table 4). One possibility might be that increased PA production in both mutants is due to a reduction in PA degradation rather than an increment in its synthesis, since deletion of *rcsB* resulted in a greater reduction of *nan* gene expression (Table 4); however, this hypothesis awaits confirmation. Nevertheless, although the Rcs pathway diminished PA production at high temperatures, it does not seem to be responsible for thermoregulated inhibition of group II PA capsules in *E. coli* at low temperatures, since no detectable PA was observed in either *rcsA* or *rcsB* mutants.

Lastly, we further investigated the possible role of RcsB controlling the expression of other molecules implicated in the transcriptional control of *cps* and/or *kps* operons. It has been shown that RcsB auto-regulates the Rcs system by modifying the expression of *rcsA* [33] and *rcsD* [42] genes. Thus, we found that RcsB greatly enhanced *rcsA* expression at both temperatures (Table 5). RcsB also affected *rcsC* expression in a temperature-dependent manner (Table 5). Since RcsC can sense external signals to trigger the action of the Rcs pathway, this result would provide a mechanism by which RcsB modifies the capacity of the bacteria to perceive changes in growth temperature. In contrast, although RcsB does not seem to regulate *rcsF* expression, activation of RcsF does not require increased transcription [15].

The regulatory mechanism of *kps* operon transcription remains unclear, although recent studies have identified a set of molecules, including H-NS and SlyA, as important factors in this regulation [4,7]. Our results show that RcsB up-regulates and down-regulates the expression of all tested genes (*dsrA*, *rfaH*, *h-ns* and *slyA*) at high and low temperatures, respectively. Although more studies are required to clarify this complex regulatory mechanism, we want to emphasize some aspects. Deletion of *rcsB* repressed the expression of *rfaH*, *slyA* and *h-ns* genes at 37°C (Table 5), which may explain the decreased *kps* expression observed in the *rcsB* mutant, since all three proteins are necessary for maximal transcription of the group 2 capsule gene cluster at high temperatures [4,7]. On the other hand, *kps* transcription was repressed from PR3 at 20°C by H-NS [7]. SlyA was able to promote *kps* transcription from PR3 at low temperatures in an *h-ns* mutant; however, there was insufficient SlyA to activate transcription in WT [7]. Thus, a critical point in this regulation seems to be the relative concentration of these two proteins. We and others previously found that *slyA* expression is temperature-dependent, with a reduced expression at low temperatures [4,21]. Since *slyA* expression was slightly enhanced at low temperatures in an *rcsB* mutant, we speculate that this increment may overcome H-NS repression and explain the increment in *kps* transcription at 19°C in the *rcsB* mutant (Table 5). Furthermore, the *rcsB* mutant showed a great increase in *dsrA* expression at 19°C. dsrA is an RNA molecule in which commencement and stability of transcription are higher at low temperatures [15]. dsrA RNA binds to h-ns RNA and blocks its translation [19], which would allow SlyA to promote *kps* transcription. Recently, it was found that the heterodimer RcsB-BglJ strongly activates *leuO* transcription [43], a known antagonist of H-NS [44,45]. Thus, we propose that RcsB may repress *kps* transcription at low temperatures by tipping the balance in favour of SlyA.

Overall, these results suggest that the Rcs phosphorelay, and particularly the RcsB regulator, are global keys in the capacity of *E. coli* K92 to adapt to different environments either inside or outside host cells, and they provide a better understanding of the complex thermoregulatory network governing capsule synthesis in *E. coli*.
